# Supplementary material for: Enhancing the communication of radiation exposure data for radiological workers in Korea using data visualization techniques
Source: PLoS One. 2025 May 5;20(5):e0323091. doi: 10.1371/journal.pone.0323091 (PMC12052103; doi:10.1371/journal.pone.0323091)
Supplement: Attachment — Submitted filename: Response to Reviewers.docx [file pone.0323091.s002.docx]

**TO editor.**

Thank you for your assistance in the review process. In response to the editor's request, we have made the following revisions and improvements: **First,** we have revised the title to enhance the academic value of the manuscript and align it with PLOS ONE’s editorial policies.

**Revised Title -** **Enhancing the Communication of Radiation Exposure Data for Radiological Workers Using Data Visualization Techniques**

**Reason for Title Revision** - Based on the reviewer’s feedback, the title has been revised to align more closely with the content of the paper and to clearly highlight the research’s objectives and contributions.

**1) Appropriateness of the Revised Title**

**Highlighting Key Content**: The revised title effectively encapsulates the core of the study, which focuses on utilizing visualization techniques to improve the communication and understanding of radiation exposure data.

**2) Clarity of the Revised Title**

The title is concise while clearly specifying the study’s target audience (radiological workers), the issue addressed (radiation exposure data), and the methodology employed (visualization techniques).

**Second,** in accordance with the editor's recommendation, we have removed Figure 10, which contained map/satellite images. Additionally, we have revised and added the following content.

A map chart can be displayed on a map, enabling regional comparisons. The taller the bar, the greater the collective dose in that region. Presenting such map charts alongside tables in the annual report could significantly enhance readers' understanding.

**Thank you once again for your valuable feedback and support. I sincerely hope that the manuscript will be accepted for publication.**

**Response to Reviewer 1**

Dear Reviewer,

Thank you for your detailed and constructive feedback on our manuscript. We have carefully considered your comments and made substantial revisions to address the issues you highlighted. Below, we provide our responses to your comments and outline the specific changes made in the manuscript.

Major Concerns

(1) Lack of a Scientific Hypothesis or Research Question:

Reviewer Comment: The paper does not test a specific scientific hypothesis or research question. Instead, it largely presents a collection of visualization methods applied to existing data from the KDCA's 2022 Annual Report.

**Response:** We appreciate this observation and have revised the manuscript to clearly articulate the study’s research objectives and its contribution to the field. In the **Introduction**, we now emphasize the specific research question this study seeks to address:

*"How can data visualization techniques improve the accessibility and comprehension of radiation exposure data for radiological workers and other stakeholders?"*
We believe this provides a clearer framework for the study's purpose and relevance. Additionally, the **Conclusion** section has been revised to underscore the practical implications of addressing this question.

(2) Absence of Original Data Collection or Experimental Results:

Reviewer Comment: "The study doesn't collect new data or provide new findings about the radiation exposure trends. It uses already available public datasets and focuses on presenting them in a more accessible format."

**Response:** We acknowledge this limitation and have addressed it explicitly in the **Discussion** and **Conclusion** sections. The revised manuscript recognizes the reliance on pre-existing datasets and proposes directions for future research, including the development of real-time data collection systems and the exploration of causal relationships using primary data. These changes aim to position this study as a foundational step towards improving data accessibility and encouraging further empirical investigations.

(3) Limited Statistical Testing:

Reviewer Comment: "The paper mentions some statistical tests (e.g., regression lines for trends), but it doesn’t appear to use statistical methods to draw conclusions from the data."

**Response:** While this study focuses primarily on the application of visualization techniques, we agree that incorporating advanced statistical analysis could strengthen future work. In the revised **Discussion** section, we highlight the potential for applying multivariate regression models and machine learning algorithms in future studies to uncover deeper insights into radiation exposure trends and disparities.

(4) Limited Novelty in Visualization Techniques:

Reviewer Comment: "The visualization techniques employed, while effective, are not particularly novel. There is limited innovation in the choice of tools or in how these tools are applied to the dataset."

**Response:** We recognize the need to explore more innovative visualization methods. To address this, the revised manuscript includes a discussion of potential advancements, such as interactive dashboards, augmented reality, and dynamic visualization tools. These are outlined as future directions in both the **Discussion** and **Conclusion** sections. While this study employs well-established techniques, we believe their application to occupational radiation exposure data provides an important contribution to the field.

**(5) General comments on manuscript clarity and focus**

**Reviewer comment**: "Given that this article does not focus on scientific testing or original research, it might be more suitable for journals focusing on public health, epidemiology, or data visualization."

**Response**: We appreciate this insight and have revised the manuscript to align more closely with the goals of the journal by emphasizing the practical applications and interdisciplinary relevance of the study. The **Abstract** and **Introduction** now more explicitly highlight the broader implications of the findings for public health and safety management.

Minor Concerns

(1) Clarification of Objectives in the Abstract and Introduction:

Changes Made: Revised both sections to explicitly state the study’s objective of evaluating the effectiveness of visualization techniques.

(2) Improvement of Figures and Captions:

Changes Made: Enhanced figure captions to provide clearer explanations of the data and insights conveyed by each visualization.

(3) Discussion of Limitations:

Changes Made: Expanded the limitations section to explicitly address the absence of original data collection and the challenges associated with using R for Chernoff face visualizations.

Summary of Changes Made

Abstract: Revised to explicitly state the study’s objectives, findings, and practical implications.

Introduction: Expanded to articulate the research question and emphasize the study’s contribution to improving occupational health practices.

Discussion: Addressed limitations of the current study and proposed directions for future research, including real-time data collection and advanced visualization techniques.

Conclusion: Strengthened to highlight the practical contributions of this study and its potential global relevance in occupational health management.

We sincerely thank you for your valuable feedback, which has greatly improved the quality and clarity of our manuscript. We hope that the revisions meet your expectations and address your concerns comprehensively.

Sincerely,

Kyoungho Choi

Jeonju University

**Response to Reviewer #2**

Dear Reviewer!

Thank you for your positive and encouraging feedback on our manuscript. We are delighted to hear that you found the manuscript well-written and that the introduction, methods, results, and discussion met your expectations. Below, we respond to your specific comments.

Reviewer Comment:

"The manuscript was good, interesting, and well-written."

"Introduction was well-written and included the aim of the study."

"Materials and methods were well-designed."

"Results were well-illustrated."

"Discussion was well-written.“

**Response:**

We sincerely appreciate your kind words regarding the quality and structure of our manuscript. Your acknowledgment of the clarity in the introduction, the robustness of the methods, the visual representation of the results, and the coherence of the discussion is highly motivating. We have ensured that these strengths are preserved while addressing the comments from the other reviewer to further enhance the manuscript.

Thank you for your valuable time and positive evaluation, which has greatly encouraged us to continue refining our work.

Sincerely,

Kyoungho Choi

Jeonju University
