# Supplementary material for: Enhancing the communication of radiation exposure data for radiological workers in Korea using data visualization techniques
Source: PLoS One. 2025 May 5;20(5):e0323091. doi: 10.1371/journal.pone.0323091 (PMC12052103; doi:10.1371/journal.pone.0323091)
Supplement: Attachment — Submitted filename: Response_to_Reviewers_auresp_2.docx [file pone.0323091.s003.docx]

**Additional Editor Comments:**

I have revised the title as follows.

1. Title: Please include the state or country where the proposed method is applied. If this method specifically targets the health sciences community in a particular region (e.g., Asia), please indicate this in both the abstract and introduction.

(ans.) The need for visualizing statistical information on radiation exposure doses for medical institution radiological workers is not limited to a specific region. However, since this study utilizes data from Korea, we have explicitly included 'Korea' in the title.

**Enhancing the communication of radiation exposure data for radiological workers in Korea using data visualization techniques**

2. Images and Tables: Improve the quality of the images presented in this work. All tables must be revised; some need to be enlarged with clearer text and color.

(ans.) I have reformatted and revised the tables. And I have adjusted the image sizes to improve readability.

3. Column Titles: Highlight some column titles to improve visibility.

(ans.) I have emphasized the column titles to improve readability and have reformatted the tables.

4. Captions: Ensure all figures have appropriate captions.

(ans.) I have enhanced readability by adding detailed captions to Figures 1 through 11.

5. Capitalization: Maintain consistent capitalization for place names, using either title case or sentence case as appropriate.

(ans.) I have revised the manuscript title, section headings, and table titles to follow Title Case formatting. I have revised all place names to start with capital letters. Additionally, I have corrected the place names in Figures 9 and 10.

6. Units: Provide appropriate units for all figures and tables (e.g., year, age, crime rate).
(ans.) Where necessary, units have been inserted in parentheses in the figures.

7. Typo: Correct "Categoty" to "Category."
(ans.) I have corrected the typos.

8. Spacing: Ensure "0.38 mSv" includes a space between the number and unit.
(ans.) I have added a space between the numbers and units.

9. Line 81: Remove the quotation ("") symbol.
(ans.) I have removed the quotation ("") symbols.

10. Figure 5 Caption: Does it need to specify that it is a U.S. state map? If so, please cite the reference as well.

(ans.) I have revised the caption to clearly indicate that it is a U.S. map and have also included the source.

**Fig.4 [**U.S. state heat map illustrating murder and non-negligent manslaughter crime rates in 2012. Reder regions indicate higher crime rates. Data source: Thoplan (2014).]

**Reviewer's Comments:**

Reviewer #1

**1.** The study is focused on data from South Korea and may not necessarily be generalizable to other regions or countries. Differences in healthcare infrastructure, radiation safety regulations, and exposure practices could lead to different results in other settings. Cross-country comparisons should account for these contextual differences when making conclusions about radiation safety on a global scale.

**(ans.)** Data visualization is an extremely effective tool for disseminating information, enhancing accessibility, ensuring objective communication, and making complex data more comprehensible to the general public. While some previous studies have explored the effectiveness of data visualization in statistical communication, few have focused on methodologies specifically related to occupational radiation exposure in the healthcare sector.

This study examines various data visualization methodologies based on the **"2022 Annual Report on Individual Exposure Doses of Radiological Workers"** published in South Korea. Through this analysis, the study explores a range of visualization techniques that can enhance the effective communication of healthcare-related statistical information and emphasizes the necessity of such methods.

Although the dataset used in this study originates from South Korea, the visualization techniques discussed are not limited by regional constraints. Therefore, we believe that the findings of this study can be **generalized** beyond a specific geographical context.

2. Visualization Over-reliance:

While the article advocates for using visualization techniques to simplify complex data, there is a risk of over-relying on visualizations without ensuring that they provide enough depth for decision-making. Visualization methods like radar charts or Chernoff faces are useful for highlighting patterns but may not provide sufficient statistical rigor or details required for comprehensive scientific analysis or policy development.

(ans.) The data provided by relevant institutions is primarily presented in tabular format, containing information on radiation exposure doses categorized by occupation, region, and gender. These annual reports are not only reviewed by experts but also by the general public, including individuals with limited knowledge of radiation. However, tables can be challenging to interpret for readers with low numeracy skills, potentially leading to misinterpretations.

This highlights the **limitations of conveying information solely through tables.** Therefore, this study emphasizes the importance of enhancing readability by **complementing tabular data with appropriate visualizations** to improve accessibility and understanding.

It is important to note that **this study does not advocate for replacing tables entirely with visualizations** but rather suggests that combining both methods can enhance data interpretation. We kindly ask for an accurate understanding of this perspective.

Furthermore, during the process of generating graphs, statistical analysis tools can automatically perform statistical tests such as **analysis of variance (ANOVA)** to determine statistical significance. This ensures that the visualization process maintains **statistical rigor and reliability**.

3. Ethical and Privacy Concerns:

Although not explicitly addressed in the article, the study uses individual exposure data from healthcare workers. It is important to ensure that privacy and ethical considerations are taken into account when handling and presenting sensitive data. The article does not mention any safeguards for ensuring that the data is anonymized or that privacy concerns are adequately addressed.

(ans.) Ethical considerations are, of course, essential. However, the data used in this study is based on an annual report published by the South Korean government, and no personal information was utilized. Therefore, additional descriptions regarding data anonymization are not necessary. We kindly ask for your understanding on this matter.

Reviewer #2: The revised version of the manuscript was good written and all comments of reviewers were addressed.

(ans.) Thank you for your help with the review.
